# Inorganic salts and intracellular polyphosphate inclusions play a role in the thermotolerance of the immunobiotic *Lactobacillus rhamnosus* CRL 1505

**DOI:** 10.1371/journal.pone.0179242

**Published:** 2017-06-08

**Authors:** María A. Correa Deza, Mariana Grillo-Puertas, Susana Salva, Viviana A. Rapisarda, Carla L. Gerez, Graciela Font de Valdez

**Affiliations:** 1 Centro de Referencia para Lactobacilos (CERELA CONICET), Chacabuco, Tucumán, Argentina; 2 Instituto Superior de Investigaciones Biológicas (INSIBIO), CONICET-UNT, and Instituto de Química Biológica “Dr. Bernabé Bloj”, Facultad de Bioquímica, Química y Farmacia, UNT, Chacabuco, Tucumán, Argentina; 3 Facultad de Bioquímica, Química y Farmacia. Universidad Nacional de Tucumán, Tucumán, Argentina; National Renewable Energy Laboratory, UNITED STATES

## Abstract

In this work, the thermotolerance of *Lactobacillus rhamnosus* CRL1505, an immunobiotic strain, was studied as a way to improve the tolerance of the strain to industrial processes involving heat stress. The strain displayed a high intrinsic thermotolerance (55°C, 20 min); however, after 5 min at 60°C in phosphate buffer a two log units decrease in cell viability was observed. Different heat shock media were tested to improve the cell survival. Best results were obtained in the mediumcontaining inorganic salts (KH_2_PO_4_, Na_2_HPO_4_, MnSO_4_, and MgSO_4_) likely as using 10% skim milk. Flow cytometry analysis evinced 25.0% live cells and a large number of injured cells (59.7%) in the inorganic salts medium after heat stress. The morphological changes caused by temperature were visualized by transmission electronic microscopy (TEM). In addition, TEM observations revealed the presence of polyphosphate (polyP) granules in the cells under no-stress conditions. A DAPI-based fluorescence technique, adjusted to Gram-positive bacteria for the first time, was used to determine intracellular polyP levels. Results obtained suggest that the high initial polyP content in *L*. *rhamnosus* CRL 1505 together with the presence of inorganic salts in the heat shock medium improve the tolerance of the cells to heat shock. To our knowledge, this is the first report giving evidence of the relationship between polyP and inorganic salts in thermotolerance of lactic acid bacteria.

## Introduction

In the last decades, the food industry evinced the necessity of transforming traditional foods into functional ones by adding bioactive ingredients and probiotic lactic acid bacteria (LAB) [1, 2]. Worldwide, the demand for probiotics has been on the upswing. North America projected a Compound Annual Growth Rate (CAGR) of 11.4% on probiotic products for the period 2015–2020 [3]. Asia-Pacific showed the largest market in 2015 with US$15 billion, an increase of about 9% is expected for 2017. This phenomenon may be attributed to a larger awareness on the role of probiotics in promoting good health leading tothe demands for innovation on dried probiotic products.

The diversification of the market of probiotic foods relies on the availability of new strains or new formats of probiotic cultures. Until now, fermented dairy products, mainly fermented milks, have been used as the most successful for delivery of probiotic bacteria. Dietary supplements in powder containing spray dried probiotics is presented as a promising economic perspective; but the tolerance of the cells to heat stress is a bottleneck that remained to be overcome to reduce negative impact of the drying process on cell viability [4, 5]. Studies on the field reported an increase in stress tolerance by subjecting the cultures to sub-lethal chemical or physical stress [6–8], by using thermo-protectants (sugars and soluble fiber) or by growing the bacteria under suboptimal conditions of pH and temperature [6, 8–12]. Growth media may also induce stress tolerance. Recently, Huang et al. [13] demonstrated that hyper-concentrated sweet whey as culture media enhanced multi-stress tolerance acquisition in bacteria which may be related to the presence of polyphosphate (polyP). Other authors [14–17] reported that cytoplasmic accumulation of polyP by LAB would be a survival strategy to overcome harmful stress conditions.

*Lactobacillus (L*.*) rhamnosus* CRL 1505 (CRL-1505) is considered an immunobiotic strain and its immunomodulatory effects are well documented [18, 19]. The strain is effective in preventing respiratory and intestinal infections it is mainly used as adjunct culture in functional dairy products such as yoghurt and cheese. Daily intake of these probiotic products proved to modulate both gut and respiratory illnesses in children in an advantageous manner [19].The aim of this work was to study the thermotolerance of CRL-1505 as a way to improve the survival rate at industrial scale spray drying process.

## Materials and methods

### Microorganism and culture conditions

*L*. *rhamnosus* CRL 1505 was obtained from the culture collection of Centro de Referencia para Lactobacilos (CERELA-CONICET, Tucumán, Argentina). The strain was grown for 20 h at 37°C, free pH, without agitation in MCM broth with the following composition (g/L): 0.038 MnSO_4_ (0.25 mM), 0.05 MgSO_4_ (0.42 mM), 5.6 KH_2_PO_4_ (41 mM), 3.6 Na_2_HPO_4_ (25 mM), 5.0 peptone, 10 yeast extract, and 20 lactose.

### Heat stress assay

Cells of CRL-1505 were harvested by centrifugation at 9790 x g (15 min, 4°C) and suspended in sterile potassium phosphate buffer (10 mM, pH 7.0). Bottles containing 50 mL of potassium phosphate buffer (10 mM, pH 7.0) were inoculated (2%, v/v) with the cell suspension and settled at different temperatures: 37°C (control), 50°C, 55°C, 60°C and 65°C. At 37°C no changes in cell viability was observed. Samples (1 mL) were taken after 1–5 min, and after 10, 15 and 20 min incubation at each temperature and immediately cooled in ice bath for colony counts on MRS agar by the drop count technique [20]. The plates (duplicate assays) were incubated at 37°C for 48 h. Results were expressed as the mean of log cell counts for each temperature tested.

### Effect of heat shock media composition

The thermotolerance of the strain was evaluated at 60°C for 5 min in different heat shock media: sterile potassium phosphate buffer (10 mM, pH 7.0, negative control), MRS broth (pH 6.50) (Britania, Argentina), 10% (w/v) skim milk (pH 6.70) (Milkaut, Argentina) and MCM broth (pH 6.40). From MCM broth, new heat shock media were obtained by grouping the components of the medium as follows: carbon source (MCM1), protein source (MCM2), inorganic salts (MCM3), phosphate salts (MCM4) and sulfate salts (MCM5). Samples taken were immediately cooled on ice bath for cell counting as described. Results indicate the loss of cell viability and were expressed as Δlog CFU ml^-1^ (difference of log CFU ml^-1^ after and before the heat shock).

### Effect of the growth medium composition on polyP accumulation

The effect of the growth medium on the thermotolerance and the intracellular accumulation of polyP granules in CRL-1505 was studied. Cells were grown in MCM broth with and without addition of inorganic phosphate, at 37°C for 20 h under static and free pH conditions.The cells were harvested (9790 x g, 15 min, 4°C) and inoculated (2% v/v) in the following heat-shock media: phosphate buffer (negative control) and MCM3. The cell viability before and after heat shock was determined by cell counts and flow cytometry.

### Flow cytometry analysis

Samples of CRL-1505 were stained with a cell viability kit (BD™ Biosciences, USA). Dual staining of the cells provides information of the cell membrane functionality, e.g., the retention of thiazole orange (TO) reveals themembrane integrity, a parameter of cell viability; while the accumulation of propidium iodine (PI) indicates damaged plasma membranes [21]. The cells were suspended in phosphate-buffered saline solution to approximately a concentration of 1 x 10^6^ cells ml^-1^ and incubated at room temperature for 5 min with 420 μM for TO and 48 μM for PI. Live untreated cells (37°C for 5 min condition) and dead cells (60°C for 5 min condition) of *L*. *rhamnosus* CRL 1505 in potassium buffer were used as positive and negative controls, respectively. Samples were analyzed with a FACS Calibur flow cytometer (BD™ Biosciences, USA) using two different wavelengths, 488 nm and 635 nm. The BD FACS flow was used as sheath fluid and 10000 events per sample were counted. Assays were carried out by duplicate, and data analysis was performed using low FlowJo Version 7.6.1. Results were expressed as the percentage of live, injured, and dead cells.

### Transmission electron microscopy (TEM)

Cells subjected to heat shock (60°C, 5 min) in the different media were observed with electron microscopy. For thin sectioning, the cells were fixed for one week in Karnovsky's solution: 2.66% formaldehyde, 1.66% glutaraldehyde and sodium phosphate buffer (0.1 M, pH 7.4) [22]. After wards, cells were washed three times with sodium phosphate buffer and post-fixed in 2% osmium tetroxide in the same buffer at 4°C overnight. Samples were dehydrated in ethanol series and embedded in Spurr resin. Sectioning was carried out with Potter Blum MT1 ultramicrotome. Slices were stained with lead citrate and uranyl acetate [23]. Preparations were examined with a Zeiss EM109 electron microscope (Carl Zeiss NTS GmbH, Oberkochen, Germany). Electron micrographs were collected at a magnification of × 50,000 and× 80,000.

### Measurements of polyP level

Intracellular polyP was determined in cell suspensions of CRL-1505 under different conditions. A DAPI (4´, 6-diamidino-2-phenylindole)-based fluorescence approach [24] was used. The method was adapted from the technique reported by Schurig-Briccio et al. [25] and Grillo-Puertas et al. [26] for Gram-negative bacteria. Briefly, 17 mM DAPI (Sigma) were added to cells washed and suspended in buffer T (100 mM Tris-HCl, pH 8) together with sodium dodecyl sulfate (SDS) and chloroform for cell permeabilization at an OD 600 nm = 0.02. After 15 min agitation at 37°C, the DAPI fluorescence spectra (excitation, 415 nm; emission, 450 to 650 nm) were recorded using an ISS PCI spectrofluorometer (Champaign, IL). Fluorescence (in arbitrary units) of the DAPI-polyP complex at 550 nm was used as a measure of intracellular polyP since fluorescence emissions from free DAPI and DAPI-DNA are minimal at this wavelength [24]. A non-polyP producing strain was used as negative control of polyP-DAPI fluorescence.

### Statistical analysis

Data obtained corresponded to at least three independent assays, and are reported as mean values with standard deviation. ANOVA and DGC (Di Rienzo, Guzmán and Casanoves) analysis were performed by using InfoStat (2008) software. Differences were considered significant at *p* ≤ 0.05.

## Results

### Intrinsic thermotolerance of *L*. *rhamnosus* CRL1505

**Fig 1:**
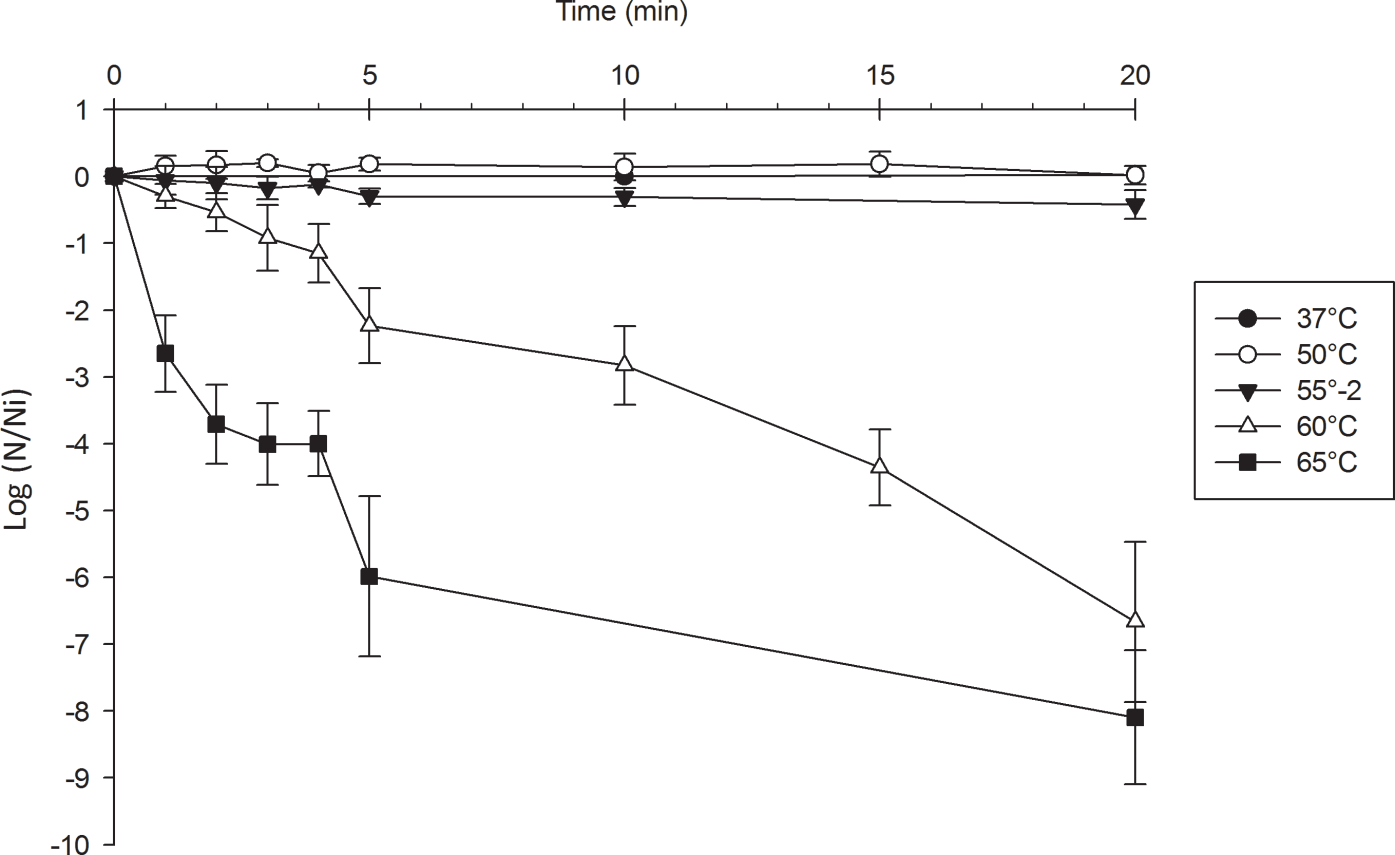
Survival of *L*. *rhamnosus* CRL 1505 heated at different temperatures in potassium phosphate buffer 10mM pH 7.

The tolerance of the strain to heat shock was evaluated in potassium phosphate buffer at different temperatures. Results are presented in Fig 1. The cell viability remained unchanged after 20 minutes at 50°C and 55°C while a decrease of 2.24 ± 0.56 and 5.98 ± 1.20 log N/Ni at 60°C and 65°C, respectively, after 5 minutes was observed. No survivors were detected at 65°C after 20 minutes.

From these results, the heat shock conditions were established as 60°C for 5 min, obtaining a value of ~ -2 for Δlog CFU ml^-1^ (difference of log CFU ml^-1^ after and before heat shock).

### Effect of heat shock media

CRL-1505 suspended in the different stress media (skim milk, MRS and MCM broths) was subjected to heat shock and the cell viability compared to that obtained in phosphate buffer solution used as negative control. No differences (*p* ≥ 0.05) in cell viability loss (from -0.23 to -0.42 Δlog CFU ml^-1^) for the complex media were obtained but for buffer solution ≥ 2 log units. Thus, the use of complex media improved the cell survival after heat shock compared to buffer solution.

Since the MCM broth is used in CERELA (CONICET) pilot plant for biomass production; it was selected in this study for evaluating the effect of the medium components in the thermotolerance of CRL-1505. The new heat shock media were arbitrarily named as MCM1 (carbon source); MCM2 (protein source); MCM3 (inorganic salts); MCM4 (phosphatesalts), and MCM5 (sulfate salts). The thermotolerance of CRL-1505 in the different shock media is shown in Table 1. Similar (*p* ≥ 0.05) results were obtained for MCM (complete medium), MCM1, MCM2 and MCM3. The subtraction of sulfate salts (MCM4) or phosphate salts (MCM5) from MCM broth produced a significant decrease (≥ 1.5 log unit) in cell viability after heat stress.

**Table 1:** Viability loss (cell death in log cycles) of *L*. *rhamnosus* CRL 1505 after heat shock (60°C, 5 min) in different media.

Heat shock media	Components	Loss of viability (Δlog CFU ml^-1^)*
**Potassium phosphate buffer**	K_2_HPO_4_+ KH_2_PO_4_	-2.24 ± 0.56^b^
**MCM broth**	Complete MCM	-0.23 ± 0.34^a^
**MCM1**	Lactose	-1.02 ± 0.52^a^
**MCM2**	Yeastextract +peptone	-0.20 ± 0.01^a^
**MCM3**	Na_2_HPO_4_+ KH_2_PO_4_+ MgSO_4_+ MnSO_4_	-0.42 ± 0.09^a^
**MCM4**	Na_2_HPO_4_+ KH_2_PO_4_	-1.74 ± 0.66^b^
**MCM5**	MgSO_4_+ MnSO_4_	-2.85 ± 0.20^c^

^*^Δ log CFU ml^−1^: difference of logCFU ml^-1^ after and before heat shock.

^a, b, c^ Data with the same superscript letter are not significantly different (*p ≥* 0.05)

In addition the cell viability of CRL-1505 after heat shock in the different media was assessed by flow cytometry. For the assay, MCM3, MCM4 and MCM5 were selected. Phosphate buffer and MCM broth were used as negative and positive control, respectively.

**Fig 2:**
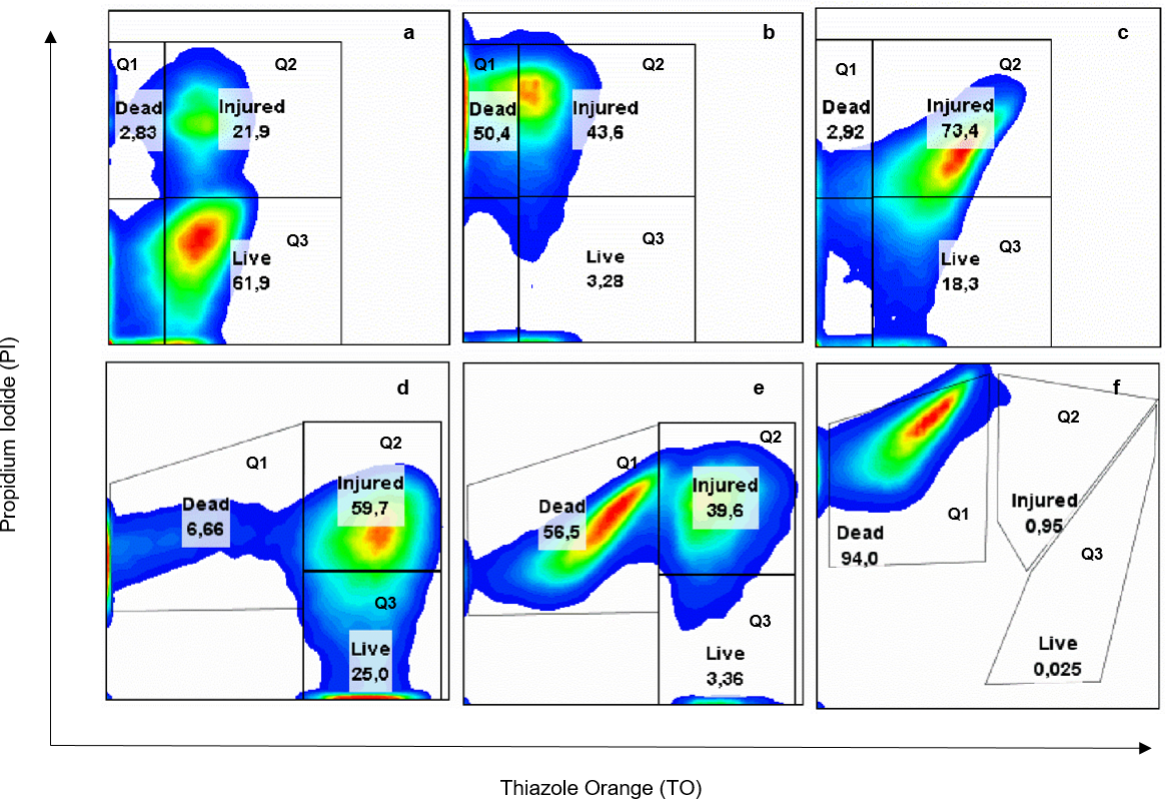
Flow cytometry fluorescence density-plot analysis (Q_1_ dead, Q_2_ injured and Q_3_ live cell population) of *L*. *rhamnosus* CRL 1505 grown in MCM broth and harvested at stationary phase of growth. Untreated cells (maintained at 37°C for 5 min) in potassium phosphate buffer (a) and after heat shock in different media: potassium phosphate buffer (b), MCM broth (c), MCM3 (d), MCM4 (e) and MCM5 (f). Values are the average of at least two experiments.

According to the results obtained, the cell population was grouped into three quadrants as it is illustrated in Fig 2: dead cells (Q_1_), injured cells (Q_2_) and live cells (Q_3_). After heat shock, the cell viability in phosphate buffer, MCM4, and MCM5 was low (3.28; 3.36 and 0.025%, respectively) (Fig 2B, 2E and 2F) compared to MCM broth (18.3%) and MCM3 (25.0%) (Fig 2C and 2D). The low cell viability obtained in media MCM4 and MCM5 by flow cytometry showed a good correlation with the number of colony forming units (CFU ml^-1^) obtained by plate count. The damaged cells population was high in all media tested except for MCM5 in which the almost whole population was dead after heat shock. Injured cells in media MCM and MCM3 were able to recover under proper growth conditions.

The results obtained by flow cytometry and plate count enabled to select a medium containing only inorganic salts as heat shock medium (MCM3), which was successful for improving the cell viability of CRL-1505 strain.

In addition, heat shock also affected the cell size and cell complexity. Morphological changes were evidenced by light scattering (S1 Fig).

### Transmission electron microscopy (TEM)

**Fig 3:**
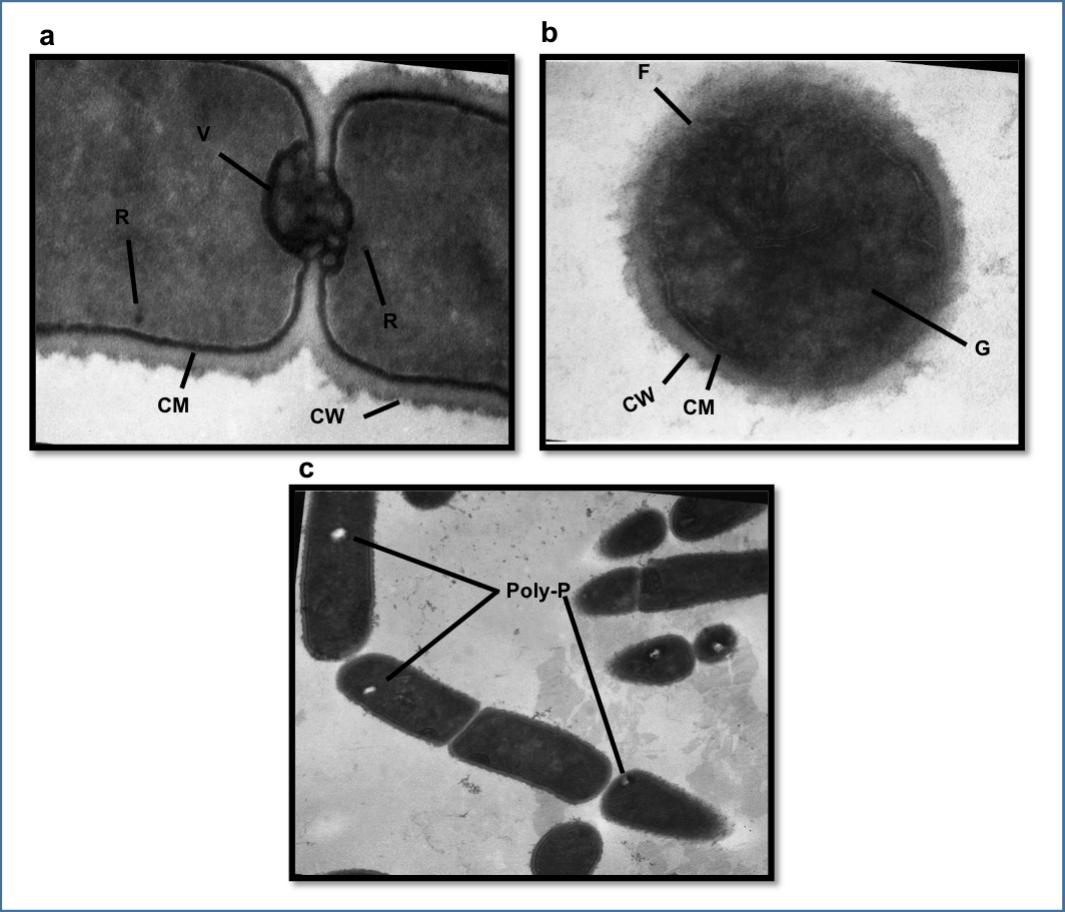
Transmission electron micrographs (TEM) of thin sections of cells of *L*. *rhamnosus* CRL 1505. The strain was maintained at 37°C for 5 min (a) and exposed to heat shock (b). V: membranous vesicles R: ribosomes CM: cytoplasmic membrane CW: cell wall F: fractures G: granular background in the cytoplasm, magnification of × 80,000. Presence of polyphosphate granules (PolyP) in the strain, magnification of × 50,000 (c).

**Fig 4:**
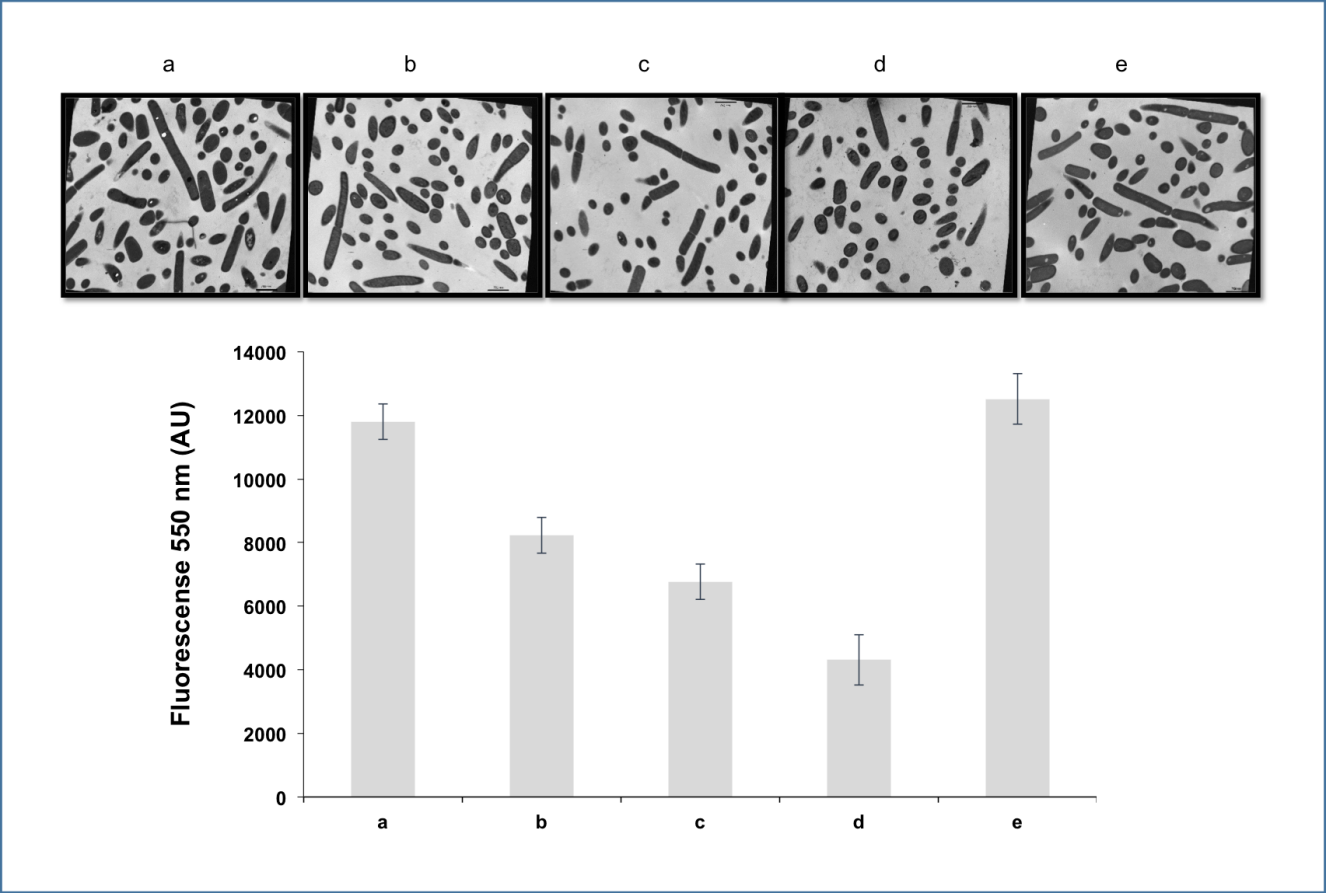
Electron micrographs (magnification of × 30,000) and polyP levels (AU, arbitrary units) of *L*. *rhamnosus* CRL 1505. The strain was maintained at 37°C for 5 min (a) and thermally stressed (heat shock) in different media: phosphate buffer (b), MCM3 (c), MCM4 (d) MCM5(e).

The morphological changes of the strain CRL-1505 subjected to heat shock in the different stress media were observed with transmission electron microscopy. Fig 3B shows the cell structural modifications post heat-shock compared to cells maintained at 37°C for 5 min (Fig 3A). Both figures were maginificated to × 80,000. In the cytoplasm of the latter, membranous vesicles and ribosomes are observed, which disappeared after heat shock. In addition, after heat shock the cytoplasmic membrane showed fractures and the cytoplasm exhibited a characteristic granular background due to the precipitation of proteins and other cellular components due to the temperature. In the cell wall, no visible changes were observed. Identical structural changes were observed for all media evaluated in concordance with flow cytometry results. Cells maintained at 37°C displayed many circular and oval holes in the cytoplasm (Fig 3C and 4A) with diameters ranging from *ca*. 50 to 200nm. The number of holes decreased after heat shock regardless the stress media except for MCM5 containing MgSO_4_ and MnSO_4_ (Fig 4E). These holes are characteristic in bacteria accumulating polyP as cytoplasmic inclusions [13, 15].

### PolyP determination after heat stress

Electron microscopy revealed the presence of polyP granules in the cytoplasm CRL-1505. Other reports have documented the presence of these polyP granules in lactobacilli by microscopy observations using Neisser staining or DAPI staining [13, 15]. In this work, the level of polyP was determined for the first time using a semi-quantitative DAPI-based fluorescence method. Since, this method has been used for Gram-negative bacteria, the method was adapted for Gram-positive bacteria. Cells of CRL-1505 (cells grown in MCM broth after 20 h of fermentation at 37°C) were first permeabilized with SDS and chloroform to allow the probe’s entry. Then, the mixture cells-probe was incubated under agitation for different periods of time that ranged from 5 to 20 min. The incubation time was fixed to 15 min since after this period of time the fluorescence released due to the complex probe-polyP did not exhibit an increase. S2 Fig (red line) shows fluorescence spectra of the DAPI-polyP complex in cells of CRL-1505 after 15 min.

The polyP levels in cells after heat shock are shown in Fig 4. On the whole, a decrease in the level of polyP was observed except for cells in MCM5 (containing only MgSO_4_ and MnSO_4_), which showed polyP levels similar to cells maintained at 37°C for 5 min, in agreement with electron microscopy observations.

### Effect of the growth medium composition on polyP accumulation

**Fig 5:**
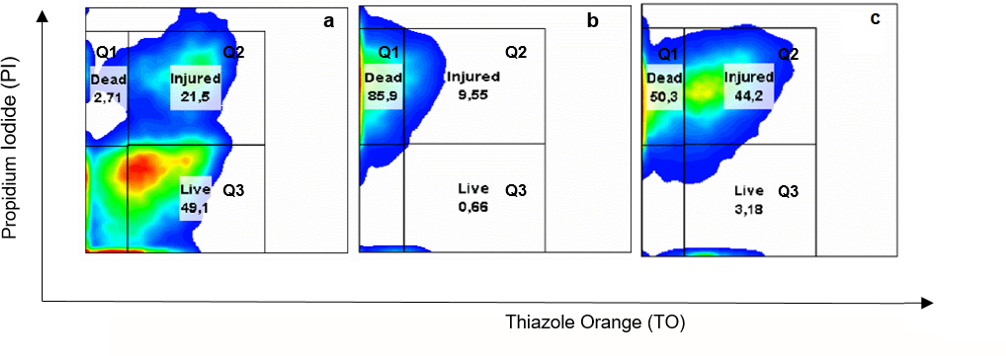
Flow cytometric fluorescence density-plot analysis (Q_1_ dead, Q_2_ injured and Q_3_ live cell population) of *L*. *rhamnosus* CRL 1505. The strain was untreated (maintained at 37°C for 5 min) (a) and thermally stressed (heat shock) in phosphate buffer (b) and MCM3 (c). Values are the average of at least three independent assays.

In order to determine the relationship between polyP and the thermotolerance of the strain, the inorganic phosphates were subtracted from the culture medium. It is known that the polyP levels in the *Escherichia coli* stationary phase cells depends on the inorganic phosphate concentration in the growth medium [25]. Thus, CRL-1505 was grown in MCM broth with and without addition of inorganic phosphates. No significant differences (*p* ≥ 0.05) in cell growth was observed but the polyP levels in cultures grown without phosphate after 20 h fermentation at 37°C were lower than those grown in MCM with phosphate (S2 Fig). The subsequent exposure to heat shock of these cells with low polyP content resulted in greater lost in cell viability than the results previously described in Table 1 (CRL-1505 with high polyP content), regardless the heat shock medium used. The lost in cell viability was 3.08 ± 0.30 and 3.25 ± 0.44 Δlog CFU/mL in phosphate buffer and MCM3, respectively. These results were confirmed by flow cytometry (Fig 5). CRL-1505 with low polyP content and exposed to heat shock in phosphate buffer displayed 85.9% dead cells (Fig 5B) while 50.4% when cells were grown in complete MCM broth (Fig 2B). Similar results were observed in MCM3, higher percentages (60.3%) of dead cells were evidenced (Fig 5C) respect to the values showed in Fig 2D (6.66%).

From these results we conclude that the thermotolerance of CRL-1505 strain was dependent on the phosphate concentration in the culture media and, consequently, on the initial levels of polyP, independently of heat shock media.

## Discussion

In this work the thermotolerance of the immunobiotic strain *L*. *rhamnosus* CRL 1505 was studied since cellular damage by heat stress is one of the major disadvantages of industrial dehydration processes such as spray drying [27–29]. CRL-1505 strain is widely used by dairy SMEs (small & media enterprises) as social probiotic in functional dairy products for undernourished children in Tucumán, Argentina. The immunobiotic strain displayed a high intrinsic thermotolerance (20 min at 55°C) in phosphate buffer without changes in cell viability, one log unit decrease after 3 min at 60°C being observed. Authors [30] reported similar thermotolerance for *L*. *plantarum* CIDCA 83114 and *L*. *kefir* CIDCA 8348 subjected to comparable heat stress conditions but using skim milk as heat shock medium. It is known that complex media are able to protect the bacterial cells under stress conditions [8]; thus, it is expected a good performance of *L*. *rhamnosus* CRL 1505 to spray drying process if complex drying media are used. In fact, the cell survival in skim milk, MCM broth and MRS broth as shock media was greater than in potassium phosphate buffer. No differences in cell viability was obtained for the complex shock media evaluated in contrast to authors reporting higher protection with skim milk [8].

Sugars are frequently added as heat protectants to drying media (solid support) in the production of dried starter cultures since they improve the survival rate of the cells during processing and further storage [31]. The retail price for sucrose (about US$ 1.45/L), which is used at 10% (w/v), impacts on the cost of bulk starter production. In this study, we formulated the medium MCM3 containing only inorganic salts as heat shock medium, which was successful for improving the cell viability of CRL-1505 strain when cultured in MCM broth containing a high phosphate concentration. These results are interesting from a technological point of view considering the price of phosphatesalts (about US$ 0.40/L) and the possibility of being used as thermoprotectant during spray drying.

Results of flow cytometry showed a large number of injured cells in CRL-1505 subjected to heat stress, which may be further recovered ascultivable cells under proper conditions. In addition, new insights on the immunobiotic strain CRL-1505 revealed that non-viable cells (heat-killed) or their cellular fractions (cell wall, peptidoglycan and exopolysaccharides) maintain the effect on the immune system and might be used in novel bioproducts as mucosal immunomodulators [32, 33]. Although damaged cells may represent an active and beneficial fraction of the microbial population, high cell viability is important for achieving the maximum probiotic effect when administered to the host [34].

TEM observations of the strain CRL-1505 showed the presence of holes in the cytoplasm of untreated cells. These holes were similar to those reported by Huang S [13] and Alcántara C [15] as polyP inclusions, which are electron-dense granules that fade under the electron beam; these granules are usually chipped or torn out during the preparation of thin cell sections leaving characteristic holes behind [35, 36]. These reports have documented the presence of polyP granules in lactobacilli using non-quantitative methodologies [13, 15]. In the present work, the level of polyP was determined and adapted for the first time for Gram-positive bacteria using the semi-quantitative DAPI-based fluorescence method, commonly used for Gram-negative bacteria. *L*. *rhamnosus* CRL 1505 growing to stationary growth phase in MCM broth with high phosphate concentrations showed a polyP level of *ca*. 13.000 AU.

In lactobacilli, the polyP accumulation is variable depending on the species, the strain considered, and the culture conditions [15]. The presence of inorganic phosphate in the culture medium is required for polyP accumulation. Huang et al. [13] demonstrated that *Propionibacterium freudenreichii* exhibits a lower amount of polyP granules when growing in sweet whey (containing about 6 mM inorganic phosphate) compared to hyper concentrated sweet whey (36 mM inorganic phosphate). Likely, Schurig-Briccio et al. reported high levels of polyP in stationary phase cultures *of Escherichia coli* growing with phosphate concentration > 37 mM, a fact that improved the stress response of the cells [25, 37].

In the present work, the level of polyP decreased by 38% when growing CRL-1505 strain in MCM broth without the addition of inorganic phosphate. However, accumulation of polyP is still observed in these cultures probably due to the presence of trace amounts of inorganic phosphate in some components of MCM broth like peptone and yeast extract.

Studies on polyP inclusions were mainly carried outin eukaryotes and Gram negative bacteria, e.g., *E*. *coli*, where they play different roles. In bacteria, the main biological function of polyP is related to substitution of ATP in kinase reactions, and -to our special interest- the physiologic adaptation of the cells to survive and growth under changing environments due to stress and deprivation [38]. In contrast, only few studies regarding polyP and stress response in LAB are available. We observed a 36% to 53% reduction in polyP level when cells of CRL-1505 with high polyP content were exposed to heat shock in the media: buffer, MCM broth, MCM3 and MCM4. These results may be related to the enzymes polyphosphates kinase (PPK) and exopolyphosphatase (PPX) which are responsible for the synthesis and degradation of polyP, respectively. Since the genes encoding for both enzymes (*ppk* and *ppx*) are under the regulation of a single operon [39] a cross regulation is expected. Authors reported that PPK is inducible by phosphate, as well as the expression of PPX [25, 40]. This fact would explain why CRL-1505 subjected to heat stress in MCM5 media (containing only MgSO_4_ and MnSO_4_) was not able to degrade polyP. In contrast, in MCM3 and MCM4 media, where the phosphate salts were present, the polyP level was reduced, although the cell viability was four times lower in MCM4 than MCM3. Therefore, the polyP degradation after heat treatment in the presence of inorganic salts (MCM3) would be related to the thermotolerance of the CRL-1505.

Based on the results obtained in this study it is assumed that a high polyP intracellular content of the cells and the presence of inorganic salts (MnSO_4_, MgSO_4_, KH_2_PO_4_ and Na_2_HPO_4_) in the heat-shock media are necessary for enhancing the thermotolerance of the immunobiotic strain CRL-1505. It is known that the accumulation of polyP provides a powerful protection against the main oxidative stress in bacteria [16, 17, 38 and 41]. The mechanism/s by which polyP affects the oxidative stress response of the cells is still unclear but it might involve the participation of gene expression regulation [42–44], protein turnover [45], polyphosphate–metal ion interactions and inorganic chaperone activity, which prevents aggregation of damaged proteins [46–48]. These multiple pathways could also protect (directly and indirectly) the bacteria from heat stress. Besides, manganese and magnesium could act as cofactors in enzymatic reactions associated to the stress response as they are involved in many enzymatic reactions in LAB [46, 47]. The polyP may play a role by interacting with these metal ions, thus improvingthe tolerance of bacteria to oxidative stress.

To our knowledge, the main contribution of this article is the relationship existing between the level of polyP inside the cells, the presence of inorganic phosphate salts in heat shock medium, and the thermotolerance in lactobacilli. Studies on the mechanism involved in the phenomenon and the practical application to spray drying process are ongoing.

## Supporting information

S1 Fig Changes in cell size during heat stress observed by flow cytometry. FSC (forward scatter) histogram of cells of CRL-1505 suspended in phosphate buffer at 37°C (blue line) and 60°C (red line).
(TIFF)

S2 Fig DAPI-polyP complex fluorescence in cells of *L*. *rhamnosus* CRL 1505. Fluorescence emission spectra of DAPI-polyP were measured in stationary cells grown in complete MCM broth (red line) and MCM broth without addition of phosphates (blue line). Green line represents a non-polyP producing strain. Data are representative of results of at least three separate experiments. AU, arbitrary units.
(TIFF)
