# Supplementary figures and images for: Inorganic salts and intracellular polyphosphate inclusions play a role in the thermotolerance of the immunobiotic Lactobacillus rhamnosus CRL 1505
Source: PLoS One. 2017 Jun 8;12(6):e0179242. doi: 10.1371/journal.pone.0179242 (PMC5464658; doi:10.1371/journal.pone.0179242)

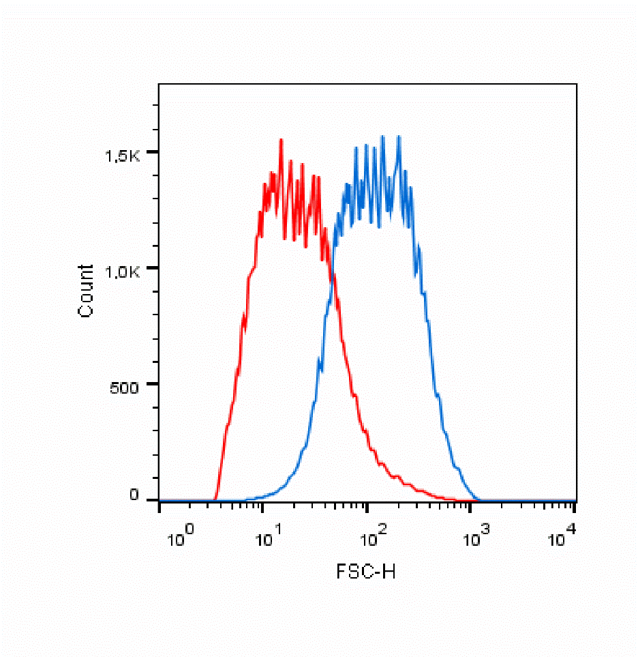

Supplement: S1 Fig — FSC (forward scatter) histogram of cells of CRL-1505 suspended in phosphate buffer at 37°C (blue line) and 60°C (red line). (TIFF) [file pone.0179242.s001.tiff]

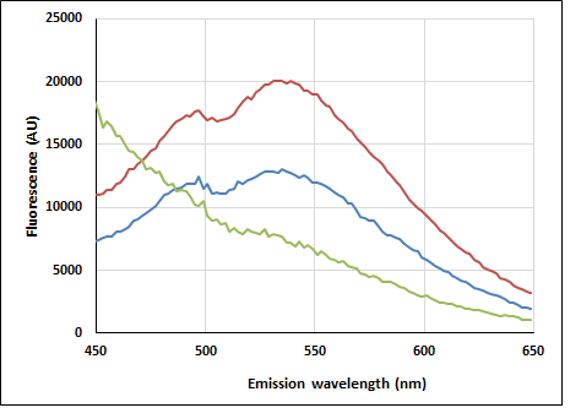

Supplement: S2 Fig — Fluorescence emission spectra of DAPI-polyP were measured in stationary cells grown in complete MCM broth (red line) and MCM broth without addition of phosphates (blue line). Green line represents a non-polyP producing strain. Data are representative of results of at least three separate experiments. AU, arbitrary units. (TIFF) [file pone.0179242.s002.tiff]
